# The impact of centralized band purchasing of pharmaceuticals on innovation of Chinese pharmaceutical firms: an empirical study based on double difference models

**DOI:** 10.3389/fpubh.2024.1406254

**Published:** 2024-07-03

**Authors:** Xinqing Chen, Shiyi Chen, Zikun Wu, Su Wang, Yuwen Chen

**Affiliations:** ^1^School of Business Administration, Shenyang Pharmaceutical University, Shenyang, China; ^2^Drug Regulatory Research Base of NMPA, Research Institute of Drug Regulatory Science, Shenyang Pharmaceutical University, Shenyang, China

**Keywords:** centralized banded purchasing of medicines, pharmaceutical companies, innovation, double-difference approach, policy effect assessment

## Abstract

In 2018, China began to gradually promote the pilot policy of centralized band purchasing of medicines. Implementing this policy has resulted in a significant decrease in drug prices. However, there needs to be a clear consensus on the impact and mechanism of action on the innovation of pharmaceutical companies. Therefore. Taking the data of Chinese Shanghai and Shenzhen A-share pharmaceutical listed companies from 2016 to 2022 as a sample, this paper empirically investigates the impact of the centralized banded purchasing policy of drugs on the innovation of pharmaceutical enterprises by using a double difference model and further analyzes the mechanism of its action. The results show that implementing the centralized banded purchasing policy can promote pharmaceutical enterprises’ innovation input and output, which is robust under the parallel trend and placebo tests. Further exploring the impact mechanism of the centralized band purchasing policy on pharmaceutical enterprises’ innovation, it can be found that it promotes innovation inputs through three channels: government subsidies, enterprise profits, and operating income. In addition, the impact of centralized band purchasing on enterprise innovation is heterogeneous in terms of region, enterprise nature, and scale. Therefore, the positive effects of the centralized band purchasing policy on promoting innovation in pharmaceutical enterprises should be fully recognized, and enterprise heterogeneity should be taken into account when implementing the policy. This study provides empirical evidence on the implementation effect of the centralized banded purchasing policy and provides lessons for continuously optimizing the policy to promote the high-quality development of pharmaceutical enterprises.

## 1 Introduction

Centralized band purchasing of medicines is a tool to reduce the price of medicines, the burden of medicines on patients, and the pressure on the national health insurance fund. Drug centralized volume purchasing originated in the early 1900s in the U.S. In 1910, the New York Hospital Authority was established, and joint hospitals and upstream institutions provided washing services to negotiate prices - the first collective purchasing organization GPO prototype was born. However, before the 1970s, GPO development could have been faster; in the United States, there have not been more significant gains; to 1962, the United States GPO organization only 10. Until the 1970s, the United States saw a rapid rise in healthcare costs and a decline in the proportion of insurance reimbursement, causing enormous economic pressure on hospitals, thus promoting the rapid development of GPO in the United States (1). According to the Healthcare Industry Group Purchasing Association (2), the number of GPOs in the U.S. has exceeded 900, with 98% of hospitals using at least one GPO. According to data released by the Healthcare Supply Chain Association (HSCA) (3) in August 2018, the total annual procurement value of GPOs in the United States is approximately $210 billion, which can reduce procurement costs for healthcare organizations by 10–18%. GPOs are a means of negotiating prices with vendors by pooling the needs of multiple healthcare organizations and signing a contract to obtain a certain amount of product for an “absolute purchase volume.” GPO is a system that brings together the needs of multiple healthcare organizations and negotiates prices with suppliers, and through contracting for “absolute purchasing volume,” obtains specific discounts on products, lowering purchasing prices and saving transaction costs for healthcare organizations, and playing a role in reducing the upward pressure on healthcare costs in the U.S. (4). Cleverley and Nutt (5) comparing GPO purchasing with the price of regular medical supplies, show that lower prices can be obtained for supplies, with savings ranging from 12 to 26%, affirming the importance of GPO as a way to reduce purchasing costs and reduce costs. -26% range, affirming the effectiveness of GPOs. The significant cost savings of GPOs in U.S. national healthcare spending are further highlighted in a study by Schneller (6), which estimates that GPOs save the U.S. healthcare industry $36 billion annually, as well as more than $2 billion related to human resources uncommitted to the procurement process. In addition, in India, the government’s Centralized Purchasing Agency (CPA) adopts a “double-envelope” approach to examine the quality and price of manufacturers through the demand for medicines on the Essential Medicines List (EML), which is submitted to them by hospitals and other health departments every 4 months, and only those that meet the quality requirements are allowed to bid on price. This system ensures the quality of medicines and controls the price of medicines, making the purchase price of some medicines drop significantly after scholars estimate that bulk purchasing can save the Indian government 30% of the annual expenditure on medicines (7). In the U.K., the Commercial Medicines Unit (CMU) of the U.K. Department of Health is responsible for the procurement of generic drugs for use in public hospitals through competitive bidding and procurement, and ultimately, the enterprise with the lowest price and qualified quality of the drugs wins the bid. The CMU signs a framework agreement with it to specify the winning price, the quantity of the drugs purchased, and the supply mode. The procurement of medicines by public hospitals only accounts for 20 to 30% of the expenditure on medicines of the National Health Service (NHS) in the U.K. Although the procurement quantity of generic medicines exceeds half of the total, their amount only accounts for 20%. To a certain extent, the government-led centralized banded purchasing of medicines in the United Kingdom has reduced the price of medicines and improved the utilization rate of NHS funds (8).

For a long time, China’s pharmaceutical industry has been suffering from the problems of high drug prices and unregulated circulation, which aggravate the burden of patients’ medication, and the government is facing the pressure of reducing medical expenditure and improving the utilization rate of medical resources. In 2018, the state adopted and began to gradually promote the pilot centralized band-volume procurement of medicines, with the general idea of “state organization, alliance procurement, platform operation” and the main principles of “band-volume procurement, volume-for-price, volume–price linkage, recruitment and procurement in one package, ensuring the volume of use, and guaranteeing the return of money” (9, 10). By government departments, the number of drugs used by each public medical institution is collected in a unified manner for consolidation, and through bidding and bidding, an apparent procurement volume is used to attract multiple enterprises to participate in the bidding, and enterprises participating in the centralized purchasing of medicines in quantities are required to meet the State’s standards. The centralized procurement of drugs organized by the State sets a high threshold for drug manufacturers, and the bidding drugs must be original drugs and generic drugs that have passed the consistent evaluation of quality and efficacy of generic drugs by the State Drug Administration to ensure the therapeutic effect. Finally, the supplier with the lowest offer is selected to sign the procurement contract. According to statistics from the National Health Security Bureau (11), by 2020, the actual procurement volume has reached 2.4 times the agreed procurement volume, saving the health insurance fund more than 100 billion yuan in budgeted drug costs overall. According to the Central People’s Government data statistics, by May 2023, China will go from the pilot to the normalization of the institutionalization of the organization to carry out the centralized procurement of 333 kinds of drugs covering anti-infective, cardiovascular and cerebrovascular diseases, anti-allergy, mental illness and other common diseases, chronic diseases, and medicines, and the price has dropped significantly, on average, more than 50%. The centralized purchasing of medicines significantly reduced the price of medicines, the patient’s financial burden and the burden of medication significantly reduced, improved accessibility of medicines; patients are the most direct and largest beneficiaries (12); set the “threshold” to ensure the quality of medicines for patients, and promote the high-quality development of the pharmaceutical industry; effectively slow down the pressure on the health insurance fund, and improve the quality of medical resources and health insurance funds. At the same time, it can improve the utilization rate of medical resources and health insurance funds.

The pharmaceutical industry is a competitive field, and innovation is the core competitiveness of pharmaceutical enterprises. In the existing research on centralized volume purchasing of medicines, one viewpoint is that centralized volume purchasing of medicines enables pharmaceutical manufacturers to obtain large-scale orders through one-time centralized transactions, which can reduce the transaction costs of enterprises and enable them to spend more human and material and financial resources on production and innovation activities. At the same time, in the economic environment of financing difficulties, high innovation costs, and shortage of innovation funds constraining the development of innovation activities and innovation ability of enterprises, the health insurance sector takes out about 30% of the total budget to pay the first medical institutions, thereby shortening the turnover period of the payback, but also for the enterprise to save money, and is conducive to enterprises to carry out innovation activities and improve innovation ability (13). Another viewpoint is that there is a considerable price difference between “awarded” and “non-awarded” medicines, and the lower price of “awarded” medicines compresses the profit margins of enterprises, which hurts their business performance and is not conducive to the improvement of their business performance. This hurts the business performance of enterprises and could be more conducive to promoting innovation and R&D investment (14, 15). In summary, there is no unified conclusion in the literature on the relationship between the innovation effect of the centralized band purchasing policy on pharmaceutical production enterprises, and the existing literature is limited to a single aspect, with few scholars having conducted complete empirical research on the centralized band purchasing policy. Considering the diversity of innovation subjects, when assessing the impact of the centralized band purchasing policy on the innovation activities of enterprises, the characteristics of their property rights, scale, and geographic location should not be ignored, and different enterprises are affected differently by the implementation of the centralized band purchasing policy, which is a key factor to be considered when assessing the effectiveness of the policy. In addition, through what mechanism does the centralized band purchasing policy achieve enterprise innovation? All of the above questions need to be answered by further research. Therefore, this paper regards the centralized band purchasing policy as a quasi-natural experiment and adopts the double-difference approach to study this viewpoint to test the real impact effect and mechanism of the centralized band purchasing policy on pharmaceutical enterprise innovation.

The possible contributions of this paper are: (i) It is the first empirical study from the dual perspectives of innovation input and innovation output of pharmaceutical enterprises, which systematically assesses the innovation effect of the implementation of centralized banded purchasing policy on pharmaceutical enterprises, and provides a more strategically valuable reference for the further implementation of the centralized banded purchasing policy, stimulating innovation of enterprises and the construction of an innovative country. (ii) This paper empirically investigates the heterogeneity of the impact of centralized band purchasing on enterprise innovation from the aspects of enterprise nature, regional distribution, and enterprise scale, which improves the horizontal dimension of the study, identifies the impact of centralized band purchasing on pharmaceutical enterprises’ innovation more precisely, and provides a favorable basis for promoting the targeted and differentiated implementation of the policy. (iii) This paper proposes three conduction paths of the drug centralized band purchasing policy affecting the innovation of pharmaceutical enterprises, discusses the mechanism of the implementation of the drug centralized band purchasing policy on the innovation of pharmaceutical production enterprises, enriches the longitudinal dimension of the study, and helps to make up for the deficiencies of the relevant literature and improve the theoretical system of the drug centralized band purchasing affecting the innovation of pharmaceutical enterprises.

The remaining parts of the paper are organized as follows: Part II, Theoretical Analysis and Research Hypotheses; Part III, Empirical Research Design; Part IV, Empirical Analysis; Part V, Heterogeneity Test; Part VI, Influence Mechanism Analysis; and Part VII, Conclusion and Implications.

## 2 Theoretical analysis and research hypotheses

The core competitiveness of pharmaceutical enterprises is mainly embodied in the ability to innovate. The neoclassical school of economics believes that enterprise innovation has the characteristics of high risk and externality, which will lead to the problem of “market failure” and need government support, which provides a theoretical basis for implementing national innovation policy and industrial policy (16). Through administrative means, the government helps enterprises overcome innovation risk and improves the supply of independent innovation in the market. The government uses policy instruments to adjust and optimize the structure of the pharmaceutical industry by implementing policies that directly or indirectly influence enterprises’ investment behavior and strategic decisions. With the existing research, most scholars believe that industrial policy has positive incentives for innovation development and total factor productivity of enterprises. For example, Feng (17) selects Shanghai and Shenzhen A-share listed companies as the research object and concludes that industrial policy has a positive and significant effect on the innovation efficiency of enterprises during the 12th Five-Year Plan period. Han and Gao (18) use the total factor productivity of A-share listed enterprises in Shanghai and Shenzhen from 2004 to 2022 as a measure of high-quality development of enterprises, construct a regression model, and carry out an in-depth analysis and testing of different nature of enterprises and different influence mechanisms. It is found that industrial policy promotes the high-quality development of enterprises through the realization of the innovation, financing, and aggregation effects, and there is a significant causal relationship between them. Wei and Cheng (19) used Made in China 2025 as a quasi-natural experiment to study and analyze the data of A-share listed companies from 2012 to 2018 using double differences. They found that industrial policy affects enterprise innovation through the signaling effect. At the same time, there are heterogeneous effects in enterprises of different natures, such as age, size, and nature of property rights. Through analysis and research, Xu (20) concluded that industrial policy has the potential to stimulate advanced production factors, which is conducive to fostering the awareness of independent innovation and motivating the innovative behavior of market players. Therefore, industrial policy makes enterprises improve their enthusiasm for independent innovation so as to increase innovation investment and promote enterprise innovation.

Compared with the previous drug policy, “quantity for price” this procurement method will force enterprises to convert their business concepts, put more energy into innovation, and enhance the strength of research and development and innovation capacity, to improve product quality and competitiveness, is the biggest highlight of the centralized quantity purchase of drugs. The introduction of centralized band purchasing of medicines is an industrial policy that aims to provide better medical protection and services for the public by reducing drug prices, improving the stability and quality of drug supply, and promoting the healthy development of the pharmaceutical industry. However, the academic community has positive and negative views on the innovation of pharmaceutical companies. On the one hand, centralized band purchasing drugs has a “positive effect” on the innovation of pharmaceutical companies (21). Pharmaceutical manufacturers must meet national standards to participate in the centralized purchasing of medicines. State-organized centralized procurement of medicines sets a high threshold for drug manufacturers, and bids must be for originator drugs and generic drugs that have passed the State Drug Administration’s consistency evaluation of the quality and efficacy of generic drugs to ensure therapeutic efficacy. As a result, pharmaceutical companies are incentivized to invest more in R&D and innovation to improve drug quality (22). At the same time, “winning” enterprises “price for volume” to obtain a larger market share can reduce the cost of goods sold and reduce financial risk, so you can have more money to invest in pharmaceutical research and development, to improve the competitiveness of enterprises to inject power (23). Tan and Fan (24) believe that the pharmaceutical industry’s policy is to reshuffle the innovative pharmaceutical enterprises to release the policy dividend, promote the quality of the pharmaceutical market efficiency, and promote the healthy development of the pharmaceutical industry. Huang and Tao (25) suggest that centralized band purchasing of medicines can promote the transformation of enterprises, facilitate the research and development of new drugs, and enhance the competitiveness of enterprises. Through case study analysis, Li and Shen (26) found that the intensity and level of research cost investment of enterprises under centralized banded purchasing of medicines are on the rise as a whole, which positively promotes the innovation of pharmaceutical enterprises. On the other hand, some scholars believe that centralized band purchasing has a “negative effect” on the innovation of pharmaceutical companies. The government negotiates with enterprises with 60% of the market share. It obtains lower procurement prices, which will financially impact enterprises and prompt them to reduce R&D investment to minimize innovation risk. Some scholars have shown that lower drug prices make it difficult for firms to reap the benefits of their R&D investments, leading to a negative impact on R&D and a reduction in R&D investment expenditures (27–29).

In summary, this paper argues that although the centralized volume purchase of medicines has reduced the price of medicines, it has expanded the market share of the winning medicines. The enterprises have ensured their profits through price and quantity by “thin profit and high sales.” Despite the drastic reduction in the price of the medicines, the increase in the sales volume has offset the negative impact, which has made the overall sales profits show an increasing trend. The overall profitability of sales is on an upward trend. Enterprises are stimulated by the marginal gains from innovation, which stimulates and promotes enterprise innovation. Accordingly, the following research hypothesis is proposed:

*H1*: The centralized band purchasing policy for medicines promotes innovation in pharmaceutical companies.

The characteristics of innovation activities, such as high investment and risk, may make enterprises’ willingness to invest in innovation low, leading to underinvestment in innovation. Centralized band purchasing of medicines is essentially an industrial policy, and Yu et al. (30) found that industrial policy can indirectly promote technological innovation of enterprises through government subsidies. Government subsidies provide direct financial support to specific projects or enterprises to compensate for the loss of their external spillovers. This can reduce the cost of enterprise innovation activities, alleviate the financial constraints faced by enterprise innovation activities, and reduce the risk of enterprise innovation.

In addition, from the point of view of the enterprise’s capital finance, high profits provide more substantial financial support for the enterprise to improve the ability to resist innovation risk, thus promoting innovation activities. Liu and Jiang (31) are investment decision perspective is that if the system can improve the marginal benefit of innovation investment, it will also indirectly increase the innovation of enterprises. In the centralized drug procurement policy, the “winning” enterprise market share reduces the cost of sales so that the enterprise’s operating costs lower, so that the profit increases, therefore increasing the marginal cost of enterprise innovation, indirectly promote enterprise innovation, but also make the enterprise has more funds to engage in research and development, technological innovation. On the other hand, as the primary responsibility for procurement and settlement, the state pays 30% of the procurement amount in advance to the medical institutions while ensuring that the settlement is completed by the end of the following month after the delivery and acceptance. The enterprise has a stable source of funds, receives timely payment back, and increases internal cash flow. Yuan and Wang (32) found that R&D and the level of internal cash holdings are in the same trend. A rise in the level of cash availability will promote a rise in R&D. Brown and Petersen (33) pointed out that firms rely extensively on cash holdings for R&D. Innovation activities have a long cycle and require a large amount of human and material resources. When enterprises have financial difficulties, they tend to invest less in projects with high returns. If an enterprise has a stable and sufficient cash flow, it can ensure that innovative activities are carried out.

Therefore, this paper argues that the centralized band purchasing policy for medicines may affect the innovation behavior of enterprises through the channels of government subsidies, corporate profits, and operating revenues, and based on this analysis, this paper proposes the hypothesis:

*H2*: The centralized band purchasing policy for medicines can promote a positive boost to innovation in China’s pharmaceutical companies by enhancing government subsidies.

*H3*: The policy of centralized band purchasing of medicines can positively promote innovation in China’s pharmaceutical companies by boosting corporate profits.

*H4*: The centralized band purchasing policy for medicines can positively promote innovation in China’s pharmaceutical companies by boosting operating revenues.

## 3 Study design

### 3.1 Sample selection and data sources

According to the industry classification of Shen Yin Wan Guo, the Shanghai and Shenzhen A-share listed pharmaceutical companies in the three major sectors of chemical pharmaceuticals, traditional Chinese medicines, and biopharmaceuticals are selected as the research object with the 2016–2022 Chinese Shanghai and Shenzhen A-share listed pharmaceutical companies. Based on the issue of data availability and accuracy, this paper excludes ST and, *ST, PT, and related data missing companies, and the sample period is 2016–2022, excluding companies listed after 2016. Finally, 132 sample companies with 924 annual observations are screened. Among them, scientific and technological data such as innovation inputs and outputs of listed companies as well as related financial data are derived from the China Stock Market and Accounting Research Database (CSMAR),^1^ and the Juchao Information Network^2^ to find the annual reports of enterprises. In order to eliminate the influence of outliers on the study, this paper has carried out the bilateral shrinkage treatment on the relevant variables through Stata16.0 software.

### 3.2 Variable selection and description

Explained variable: enterprise innovation (Innovation). In order to comprehensively test the impact of the centralized band purchasing policy of medicines on enterprise innovation, this paper explores the two dimensions of innovation input and innovation output at the same time (34). In terms of enterprise innovation input, referring to the study of Shuai et al. (35), the ratio of R&D investment to operating income is chosen to measure the innovation input indicator, and the increase in the value of this indicator means that the intensity of enterprise’s investment in innovation also increases. Enterprise innovation output, drawing on the research of Wu and Zhang (36), once the development expenditure is recognized as an intangible asset, it means that the results of the enterprise’s R & D project have been formed. Therefore, this paper selects the development expenditure recognized as intangible assets to measure the innovation output of enterprises.

Explanatory variable: centralized band purchasing policy for medicines (Treat × Post). This paper uses the centralized band purchasing policy for medicines as an explanatory variable. The release of the Pilot Program for Centralized Purchasing of Medicines by State Organizations in 2018 is used as a quasi-natural experiment, assessed by using the double-difference method, and enterprises in the list of winning enterprises in the eighth batch of centralized band purchasing of medicines from the release of the policy to 2022 are selected as the treatment group (Treat) as 1, and the other enterprises are selected as the control group as 0; Post is the policy implementation time dummy variable, with 1 for the year after the release of the Pilot Program and 0 for other years.

Controls: Drawing on the literature of Tong et al. (37), Chen et al. (38), and Mazanec (39), we control for firm size, liquidity, concentration, age, and density of capital as control variables.

Mediating variables: This paper selects government subsidies (sub), operating income (revenue), and corporate profits (profit) as mediating variables. Variables are defined as shown in Table 1.

**Table 1:** Definitions of relevant variables.

Variable type	Variable name	Variable symbol	Variable definition
Explanatory variable	Innovative inputs	Input	R&D investment/revenue
	Innovation outputs	Output	Development expenditure of which intangible assets are recognized (in millions) (in natural logarithms)
Explanatory variable	Grouping Virtual Variables	Treat	“Successful bid” takes 1, otherwise it takes 0.
	Time dummy variable	Post	Assigned a value of 0 before 2018 and 0 in 2018 and after
Control variable	Enterprise size	Size	Natural logarithm of total assets at the end of the period
	Current ratio	Liquidity	Current assets to current liabilities ratio
	Shareholding concentration	Concentration	Shareholding of the first largest shareholder in the enterprise
	Age of business	Age	Number of years the company has been listed = Year of observation - Year of IPO
	Capital intensity	Density	Non-current assets to total assets
Mechanism variables	Government subsidy	Sub	Amount of current government subsidies (in tens of millions)
	Corporate profit	Profit	Gross operating profit (profit from main business/revenue from main business)
	Revenues	Revenue	Current business receipts (in billions of dollars)

Table 2 shows the descriptive statistics of the main variables. It shows that the minimum value of innovation input of pharmaceutical enterprises in China is 0.01, the maximum value is 0.147, and the overall mean is 0.053, which indicates that innovation input fluctuates wildly among enterprises. The level of R&D investment needs to be further improved. The mean value of innovation output in the sample is 0.337, the standard deviation is 0.64, the minimum value is 0, and the maximum value is 2.178, indicating significant differences in enterprises’ innovation output in the sample period. The overall level of innovation output of Chinese enterprises is relatively low.

**Table 2:** Descriptive statistics for key variables.

Variable name	Observations	Average value	Median	Standard deviation	Min	Max
Input	924	0.053	0.043	0.036	0.01	0.147
Output	924	0.337	0	0.64	0	2.178
Size	924	22.45	22.37	0.869	21.04	24.15
Liquidity	924	2.819	2.201	2.006	0.808	8.549
Density	924	0.474	0.483	0.148	0.209	0.729
Concentration	924	31.2	28.58	12.09	12.25	56.98
Age	924	14.75	15	6.423	5	25
Sub	924	3.995	2.339	4.291	0.256	16.17
Profit	924	0.544	0.559	0.185	0.224	0.849
Revenue	924	4.45	2.671	4.654	0.545	17.42

### 3.3 Model setup

The centralized band purchasing policy for medicines launched in China in 2018 is a quasi-natural experiment. The “winning” enterprises in this policy are the experimental group, and the “non-winning” enterprises are the control group, and the following DID estimation model is constructed by applying the double-difference method to test the relationship between the centralized banded purchasing policy of medicines and enterprise innovation:


(1) $$\begin{array}{l} Innovation_{i,t}=\alpha_{0}+\alpha_{1}Post_{i,t}\times Treat_{i,t}+\alpha_{2}Post_{i,t}+\\ \alpha_{3}Treat_{i,t}+\alpha_{i}X_{i,t}+\mu_{i}+\gamma_{i}+\varepsilon_{i,t} \end{array}$$

Where i denote individual firms, t denotes year; Innovation_i,t_ represents innovation inputs and innovation outputs, respectively; the core explanatory variable Post_i,t_ × Treat_i,t_, i.e., the interaction term indicating the implementation of centralized banded purchasing of medicines (1 for the experimental group and 0 for the control group), $\alpha_{1}$ is the critical parameter, which measures the net effect of the implementation of centralized banded purchasing of medicines. If it is significantly positive, it indicates that the implementation of centralized purchasing of medicines has limited innovative inputs or outputs of innovative pharmaceutical firms, and vice versa; it indicates that the implementation of centralized purchasing of medicines has inhibited the innovative inputs or outputs of pharmaceutical firms. If it is not significant, it indicates that the implementation of centralized banded purchasing of medicines does not significantly affect pharmaceutical enterprises. X_i,t_ denotes the set of control variables for firm size, equity, capital, and other characteristics. $\gamma_{i}$ denotes individual fixed effects, $\mu_{i}$ denotes time-fixed effects, and $\varepsilon_{i,t}$ denotes a random disturbance term.

## 4 Empirical analysis

### 4.1 Benchmark regression results

The double difference method is used to test the impact of centralized band purchasing policy of drugs on the innovation of pharmaceutical enterprises; the regression results of model (1) are shown in Table 3, the regression of columns (1)-columns (4) all control the individual and time effects, while all regressions use robust standard errors. Columns (1) and (2) show the impact of centralized band purchasing of medicines on innovation investment of enterprises. The regression coefficient of the DID indicator (Treat × Post) on input in column (1) is 0.0126, which is significant at a 1% statistical level, and the coefficient after adding control variables in column (2) is 0.0135 and still significant at 1% level, which means that centralized band purchasing of medicines can significantly promote the innovation of pharmaceutical enterprises, which means that centralized band purchasing of medicines can significantly promote the innovation of pharmaceutical enterprises. This implies that centralized band purchasing of pharmaceuticals can significantly promote the innovation investment of pharmaceutical enterprises. Columns (3) and (4) show the effect of centralized band purchasing on innovation output; column (3) does not add control variables and column (4) adds control variables and finds that the regression coefficient of the dummy variable of policy shocks is 0.3566. It is significant at a 1% confidence level, which indicates that centralized band purchasing can also enhance the innovation output level of pharmaceutical enterprises. This verifies H1. In addition, by comparing the regression results of column (2) and column (4), it is found that the promotion effect of centralized band purchasing of drugs on the innovation output of pharmaceutical enterprises is more evident than that of innovation inputs, which is because the policy has made enterprises face a low-profit spatial situation, and the funds of enterprises have been affected by some impacts. However, in order to stabilize the market and maintain its core competitiveness, the pharmaceutical company seeks to diversify its innovation strategy and increase its innovation output. For example, cooperation with other research institutions, academic institutions, medical institutions, or partners to share resources, knowledge, and technology to achieve common innovation goals.

**Table 3:** Benchmark regression results.

	Input		Output	
	(1)	(2)	(1)	(2)
Post×treat	0.0126^***^	0.0135^***^	0.3618^***^	0.3566^***^
	(2.9914)	(3.326)	(3.3984)	(3.3286)
Size		−0.0044		0.1006
		(−0.9971)		(1.3324)
Liquidity		0.0027^**^		−0.0075
		(2.5036)		(−0.3517)
Density		0.0713^***^		0.0202
		(4.5576)		(0.0693)
Concentration		0.0003		0.0057
		(0.8051)		(1.4357)
Age		0.0002		−0.0359
		(0.092)		(−0.8370)
Constant	0.0463^***^	0.0913	0.1574^***^	−1.8249
	(26.4249)	(0.9895)	−3.5727	(−1.0699)
			
Observations	924	924	924	924
R-squared	0.1638	0.2403	0.1533	0.1581
Number of id	132	132	132	132
Id fe	Yes	Yes	Yes	Yes
Year fe	Yes	Yes	Yes	Yes

^*^, ^**^, ^***^ indicate significant at the 10 per cent, 5 per cent and 1 per cent levels, respectively, with standard errors in parentheses.

### 4.2 Robustness check

#### 4.2.1 Parallel trend test

**Figure 1:**
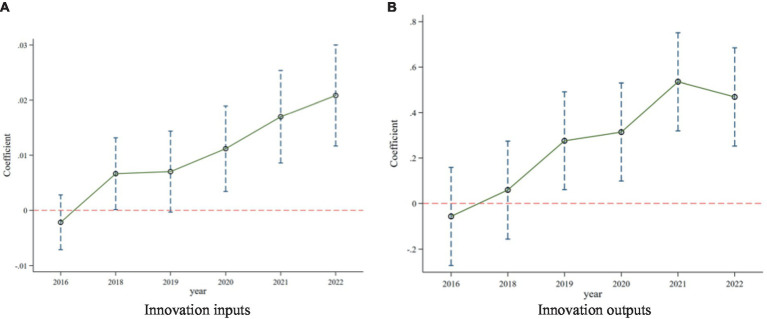
Parallel trend test results. **(A)** Innovation inputs. **(B)** Innovation outputs.

One of the core assumptions of the double-difference model, which is widely used in the assessment of policy effects, is the fulfillment of the parallel trend test, i.e., the trend of change in the experimental and control groups remained the same prior to the implementation of the policy. Based on the research methodology of Beck et al. (40), this study further tested the trend of change in the experimental and control groups, and the results are shown in Figure 1. The figure demonstrates the regression coefficients of the impact of the centralized band purchasing policy of medicines on enterprises’ innovation input and output at the 90% confidence level. From the trend of coefficient changes, before the implementation of the centralized banded purchasing policy for medicines, there was no significant difference between the winning enterprises and non-winning enterprises in terms of innovation input and innovation output indexes, while after the implementation of the policy, compared with the control group, the innovation input and innovation output enhancement of the enterprises in the experimental group rises significantly. It passes the test of parallel trend. However, the difference between innovation input in the control group and the treatment group was insignificant in 2019, which may be due to the global outbreak of the New Crown epidemic in 2019, which has caused a significant impact on enterprises. The increase in market volatility and uncertainty may cause some firms to adopt robust and conservative strategies and reduce innovation investment. Therefore, there is no significant difference between the results of the parallel trend test for 2019 between the control group and the treatment group. Therefore, the following conclusion can be drawn: before the release of the centralized band purchasing policy for drugs, Chinese pharmaceutical companies did not show significant differences in key indicators such as innovation input and total innovation output, thus passing the parallel trend test. However, after the implementation of the policy, pharmaceutical companies further increased their investment in innovation, and the number of intangible assets also showed an increasing trend.

#### 4.2.2 PSM-DID

Considering the possible interference of endogeneity problems in the sample, the propensity score matching-double difference method (PSM-DID) is used to assess the impact of the centralized banded purchasing policy of medicines on the innovation inputs and outputs of enterprises, drawing on the study of Han et al. (41), the control variables in the baseline regression model were used as covariates, and whether or not it belonged to the centralized band purchasing policy for medicines was used as the dependent variable in the Logit regression, and then, based on the propensity score obtained in the regression, the “caliper nearest-neighbor matching” or “radius matching” method was used to match the propensity score for each enterprise in the experimental group. Then, according to the propensity score value obtained from the regression, we use the “caliper nearest neighbor matching method” or “radius matching method” to find the control group samples of the same year for each experimental group; then, we eliminate the unsuccessful matched samples and carry out the balanced test; finally, the matched samples are merged and processed, and then we carry out the double-difference regression test. The estimation results are shown in Tables 4, 5. It is found that regardless of the matching method, the coefficient of Post×Treat is still significantly positive at the 1% level, which is consistent with the conclusion of the benchmark regression that the centralized banded purchasing policy of medicines has a significant positive impact on both the innovation inputs and outputs of enterprises, and it once again verifies the hypothesis H1.

**Table 4:** Innovation inputs - PSM-DID regression results.

Variant	Input			
	Caliper nearest neighbor matching		Radius match	
	(1)	(2)	(1)	(2)
Post×treat	0.0140^***^	0.0144^***^	0.0131^***^	0.0136^***^
	(3.26)	(3.41)	(3.08)	(3.32)
Size		0.00		(0.00)
		(0.17)		(−0.7398)
Liquidity		0.0025^**^		0.0026^**^
		(2.47)		(2.34)
Density		0.0670^***^		0.0712^***^
		(4.63)		(4.24)
Concentration		0.00		0.00
		(0.41)		(0.76)
Age		0.00		0.00
		(0.03)		(0.28)
Constant	0.0501^***^	(0.01)	0.0468^***^	0.06
	(26.24)	(−0.1474)	(26.70)	(0.68)
			
Observations	692	692	907	907
R-squared	0.20	0.25	0.17	0.24
Number of id	132	132	132	132
Id fe	yes	yes	yes	yes
Year fe	yes	yes	yes	yes

^*^, ^**^, ^***^ indicate significant at the 10 per cent, 5 per cent and 1 per cent levels, respectively, with standard errors in parentheses.

**Table 5:** Innovation output - PSM-DID regression results.

Variant	Output			
	Caliper nearest neighbor matching		Radius match	
	(1)	(2)	(1)	(2)
Post×treat	0.3830^***^	0.3700^***^	0.3549^***^	0.3467^***^
	(3.31)	(3.22)	(3.34)	(3.24)
Size		0.04		0.12
		(0.44)		(1.51)
Liquidity		(0.02)		(0.00)
		(−0.8485)		(−0.2092)
Density		(0.12)		(0.02)
		(−0.3257)		(−0.0849)
Concentration		0.0087^**^		0.01
		(1.99)		(1.64)
Age		(0.05)		(0.03)
		(−0.7603)		(−0.6301)
Constant	0.1992^***^	(0.29)	0.1608^***^	(2.26)
	(3.80)	(−0.1270)	(3.73)	(−1.3068)
Observations	692	692	907	907
R-squared	0.18	0.18	0.16	0.16
Number of id	132	132	132	132
Id fe	Yes	Yes	Yes	Yes
Year fe	Yes	Yes	Yes	Yes

^*^, ^**^, ^***^ indicate significant at the 10 per cent, 5 per cent and 1 per cent levels, respectively, with standard errors in parentheses.

#### 4.2.3 Placebo test

**Figure 2:**
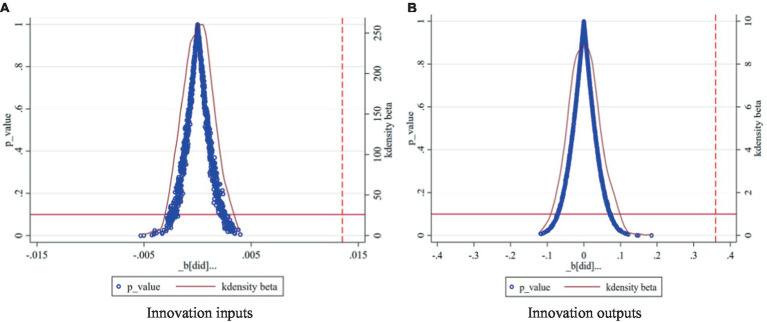
Placebo test results. **(A)** Innovation inputs. **(B)** Innovation outputs.

Given that the impact of the centralized band purchasing policy for pharmaceuticals on firms’ innovation inputs and outputs may be driven by other unobserved factors, drawing on the methodology of Zhou et al. (42), the experimental group was randomly selected for indirect testing based on the baseline model with 1,000 random replicates sampling. The distribution of *p*-values of corporate R&D investment and intangible asset values can be visualized in Figure 2. The estimates obtained by computerized random sampling of 1,000 simulated regressions are all close to the zero value, which generally shows the characteristics of normal distribution, and the vast majority of the estimates are not significant at the 5% significance level. However, the benchmark regression coefficients described earlier were 0.0135 and 0.3566, respectively, and were significant at the 1% significance level. Therefore, it can be inferred that the benchmark regression results are robust.

#### 4.2.4 Replacement of the explanatory variables

This paper replaces the explanatory variables above to avoid bias in the estimation results due to the qualitative selection of variables. It adopts the proportion of R&D investment in total assets as a proxy variable for innovation inputs, referring to the method of Wei (43), the capitalization of R&D expenditures (in billions) is selected as a proxy variable for innovation outputs, and the expenses at the development stage can only be capitalized when they meet the conditions, which is determined to be an intangible asset. The capitalization of R&D expenditures reflects the portion of expenses that can be capitalized to form the cost of intangible assets in the process of developing intangible assets in a company, which can convey the project’s success (44). The results are shown in Table 6, and it can be seen that the coefficient of Post × Treat is still significant as evidence, which indicates that the centralized band purchasing policy of drugs still plays a vital role in promoting the innovation of pharmaceutical manufacturers after replacing the measurement of the explanatory variables again and that the findings of the study are robust and reliable.

**Table 6:** Regression results - replacing explained variables.

Variant	Input	Output
Post×treat	0.0067^***^	0.2254^**^
	(4.5761)	(2.4697)
Size	−0.0025	0.3921^***^
	(−1.5060)	(3.0128)
Liquidity	0.0004	0.0346^**^
	(0.9948)	(2.1340)
Density	0.0173^**^	0.5868^**^
	(2.5673)	(2.1825)
Concentration	0.0000	−0.0009
	(0.3167)	(−0.2343)
Age	−0.0006	−0.0343
	(−0.7457)	(−1.0599)
Constant	0.0724^**^	−8.4493^***^
	(2.0091)	(−2.9525)
Observations	924	924
R-squared	0.1783	0.2432
Number of id	132	132
Id fe	Yes	Yes
Year fe	Yes	Yes

^*^, ^**^, ^***^ indicate significant at the 10 per cent, 5 per cent and 1 per cent levels, respectively, with standard errors in parentheses.

## 5 Heterogeneity tests

### 5.1 Regional heterogeneity

This paper divides the sample into eastern, central, and western regions. Further, it analyzes the impact of the centralized band purchasing policy on the innovation of enterprises in different regions. The regression results are shown in Table 7, from which it can be seen that in the eastern region, the centralized band purchasing policy can promote the innovation inputs and outputs of pharmaceutical enterprises, and it is significant at the level of 1%; for the central and western regions, the regression coefficients, although positive, are not significant, suggesting that the centralized band purchasing policy only plays a role in promoting innovation of pharmaceutical enterprises in the eastern region. Although the regression coefficient is positive for the central and western regions, the effect is not significant, indicating that the centralized band purchasing policy only promotes the innovation of pharmaceutical enterprises in the eastern region. The above phenomenon may be attributed to unbalanced regional growth in China, which is regional China’s development, which is a prominent feature in China’s economic development. The eastern region is mainly concentrated in the coastal belt and economically developed cities endowed with cutting-edge transportation, communication and energy infrastructures. The eastern region is more attractive than the central and western regions, attracting many high-quality talents who are the key to innovation; together, these factors provide solid support for innovative activities. In addition, due to the booming economy and advanced technology in the eastern region, enterprises in the region are affected by the macro environment and generally show a strong sense of innovation; these enterprises are constantly learning, updating and improving their technology. As the eastern region has unique advantages in several areas, including geography, economy and society, local governments tend to regard innovation and scientific and technological progress as their primary development strategies. The level of economic development in the eastern region tends to exceed that of the central and western regions. As a result, local governments are relatively more affluent in financial revenues. This provides governments in the eastern region with richer financial resources to fuel enterprise growth, including assistance in innovation and technological research and development. The central and western regions are usually located inland, where infrastructure development and economic levels need to catch up, and the talent pool needs to be more robust, which, to a certain extent, affects the development of the central and western regions. Therefore, the “policy effect” of” the central” ed. purchasing policy for medicines in the eastern region is particularly acute.

**Table 7:** Results of regional heterogeneity analysis.

variant	Input			Output		
	Eastern	Central	Western	Eastern	Central	Western
Did	0.0130^**^	0.01	0.01	0.4398^***^	0.11	0.20
	(2.46)	(0.81)	(1.60)	(3.35)	(0.67)	(0.87)
Size	(0.01)	0.01	(0.02)	(0.02)	0.11	0.4674^**^
	(−1.0924)	(1.42)	(−1.7141)	(−0.1931)	(1.34)	(2.36)
Liquidity	0.00	0.0029^***^	0.00	(0.03)	0.03	0.0596^*^
	(1.49)	(2.90)	(1.09)	(−1.1791)	(0.76)	(1.73)
Density	0.0696^***^	0.0787^***^	0.02	0.05	(0.36)	0.37
	(4.38)	(2.86)	(0.58)	(0.15)	(−0.4370)	(0.39)
Concentration	0.00	(0.00)	0.00	0.0099*	(0.01)	0.01
	(0.55)	(−1.6119)	(1.27)	(1.78)	(−1.0746)	(1.00)
Age	0.00	(0.00)	0.00	(0.04)	(0.11)	0.00
	(0.26)	(−0.4763)	(1.12)	(−0.7582)	(−1.1983)	(0.04)
Constant	0.15	(0.09)	0.3753^*^	0.77	(0.35)	−10.9972^**^
	(1.09)	(−0.9547)	(1.74)	(0.33)	(−0.2039)	(−2.3481)
						
Observations	545.00	225.00	154.00	545.00	225.00	154.00
R-squared	0.29	0.23	0.27	0.21	0.10	0.22
Numberofid	78.00	33.00	22.00	78.00	33.00	22.00
Id fe	Yes	Yes	Yes	Yes	Yes	Yes
Year fe	Yes	Yes	Yes	Yes	Yes	Yes

^*^, ^**^, ^***^ indicate significant at the 10 per cent, 5 per cent and 1 per cent levels, respectively, with standard errors in parentheses.

### 5.2 Firm size heterogeneity

In this study, the size of firms is categorized into two groups based on the firm’s total assets at the end of the period: those in the top three quartiles of firms’ total assets at the end of the period are considered large-scale firms. In contrast, the rest are considered small- and medium-sized firms. Table 8 reports the regression results of heterogeneity across firm sizes. The results show that in the sample of large-scale firms, the regression coefficient of the implementation of the centralized banded purchasing policy on innovation inputs is insignificant, and it has a significant positive effect on innovation outputs, which indicates that for large pharmaceutical firms, the centralized banded purchasing policy on medicines can only promote their innovation outputs. In the sample of small and medium-sized enterprises, the centralized band purchasing policy (Post × Treat) has a significant positive effect on both innovation input and output, indicating that the centralized band purchasing policy effectively promotes small and medium-sized enterprises’ innovation input and output. SMEs usually have a more homogeneous business model and product mix than large enterprises and a relatively simple management level, which allows them to adapt quickly to market fluctuations. Often, in a more competitive and resource-constrained environment, SMEs need to continuously innovate, cut costs and improve productivity to enhance their competitiveness and ensure the high quality of their products and services to gain a firm foothold and a larger market share. However, policies have had a significant impact on incentivizing the innovation output of large firms but not on their innovation inputs. Large firms usually have richer resources and more robust capabilities to carry out R&D, and they will continue to make large-scale R&D investments even without policy incentives. Innovation output, as the actual results and value of innovation activities, is an essential measure of a firm’s competitiveness and the key to its leading position in the market. By continuously launching innovative products and solutions, large companies can build strong brand recognition and loyalty in the minds of their customers. Focusing on innovation outputs ensures that innovation inputs are effectively utilized and helps companies achieve sustained competitive advantage and business growth.

**Table 8:** Results of firm size heterogeneity analysis.

Variant	Input		Output	
	Major industry	Small or medium size enterprise (SME)	Major industry	Small or medium size enterprise (SME)
Post×treat	0.01	0.0127^**^	0.5750^***^	0.1760^*^
	(1.53)	(2.40)	(2.90)	(1.82)
Size	0.01	(0.01)	(0.41)	0.1664^*^
	(1.53)	(−1.3060)	(−1.6265)	(1.70)
Liquidity	0.0063^***^	0.00	(0.02)	0.01
	(3.23)	(1.66)	(−0.2001)	(0.48)
Density	0.0432^**^	0.0694^***^	−1.7422^*^	0.5825^*^
	(2.12)	(3.99)	(−1.9551)	(1.74)
Concentration	0.00	0.00	0.01	0.00
	(0.86)	(0.62)	(0.88)	(0.21)
Age	(0.00)	0.00	(0.01)	(0.03)
	(−0.8776)	(0.33)	(−0.1078)	(−0.6896)
Constant	(0.26)	0.18	10.4058^*^	(3.49)
	(−1.3713)	(1.27)	(1.76)	(−1.6057)
Observations	278.00	646.00	278.00	646.00
R-squared	0.33	0.23	0.24	0.12
Number of id	52.00	104.00	52.00	104.00
Id fe	Yes	Yes	Yes	Yes
Year fe	Yes	Yes	Yes	Yes

^*^, ^**^, ^***^ indicate significant at the 10 per cent, 5 per cent and 1 per cent levels, respectively, with standard errors in parentheses.

### 5.3 Heterogeneity in the nature of corporate equity

Due to the difference in the nature of the enterprise’s equity, there will be differences in its organizational structure and management mode. This paper divides the sample enterprises into two categories, state-owned enterprises and non-state-owned enterprises, according to the attributes of the actual controller of the enterprises. It further tests the difference in the impact of the centralized band drug purchasing policy on innovation. The results are shown in Table 9. It can be seen that the centralized banded procurement policy of drugs has no significant effect on the innovation input of state-owned enterprises, while it improves the innovation input of non-state-owned enterprises; it has a significant promotion on the innovation output of both state-owned enterprises and non-state-owned enterprises, and the policy has a more substantial effect on the innovation output of state-owned enterprises compared with that of non-state-owned enterprises. The main reason is that state-owned enterprises pay more attention to the economic efficiency of state-owned capital, and high-risk technological innovation activities show a substantial risk aversion tendency (45). State-owned enterprises (SOEs) are usually government-owned or controlled enterprises, and national policies and strategies may influence their decisions. The national government is more concerned about the country’s overall economic stability and strategic security, so SOEs are cautious about high-risk, innovative inputs. In addition, SOEs need to bear financial, social and political responsibilities. Innovation results reflect the country’s scientific and technological development level, economic strength, and other vital indicators. The government’s intervention has led to state-owned enterprises assuming more responsibility for fulfilling social functions. Non-state-owned enterprises pursue the maximization of capital gains and sustainable development of enterprises, have greater autonomy and flexibility in R&D investment, and can quickly and keenly observe and respond to market demand. To obtain more advantages and innovation gains, non-state-owned enterprises are more willing to cooperate with the reform of national policies and improve their innovation ability. Moreover, compared with state-owned enterprises, non-state-owned enterprises face higher and more significant costs and competitive pressures in market competition, fewer policy tilts and financial subsidies, relatively more minor market shares and higher transaction costs, and pay more attention to improving their competitiveness through technological innovation to promote the continuous development of the enterprise and enhance its market position. At the same time, non-state-owned enterprises are relatively more flexible in using funds, introducing talent, etc., and can formulate corresponding R&D investment strategies according to their own development needs.

**Table 9:** Results of the analysis of the heterogeneity of the nature of the shareholding.

Variant	Input		Output	
	State-owned enterprises	Non-state-owned enterprises	State-owned enterprises	Non-state-owned enterprises
Post×treat	0.01	0.0172^***^	0.5451^**^	0.2369^*^
	(1.37)	(3.35)	(2.53)	(1.93)
Size	0.01	(0.01)	(0.01)	0.1577^*^
	(1.64)	(−1.4696)	(−0.1402)	(1.69)
Liquidity	0.00	0.0028^**^	−0.1166^**^	0.02
	(0.78)	(2.34)	(−2.1201)	(0.99)
Density	0.0359*	0.0720***	0.35	(0.01)
	(1.76)	(4.38)	(0.44)	(−0.0443)
Concentration	0.00	0.00	0.01	0.00
	(1.34)	(1.00)	(0.58)	(0.88)
Age	(0.00)	(0.00)	0.02	(0.06)
	(−0.0282)	(−0.1308)	(0.34)	(−0.9149)
Constant	−0.1995^*^	0.19	0.09	(2.94)
	(−1.7138)	(1.46)	(0.04)	(−1.3373)
Observations	259.00	665.00	259.00	665.00
R-squared	0.18	0.28	0.22	0.14
Number of id	44.00	101.00	44.00	101.00
Id fe	Yes	Yes	Yes	Yes
Year fe	Yes	Yes	Yes	Yes

^*^, ^**^, ^***^ indicate significant at the 10 per cent, 5 per cent and 1 per cent levels, respectively, with standard errors in parentheses.

## 6 Further analysis: mechanisms for the impact of centralized band purchasing policies on innovation in pharmaceutical enterprises

In this paper, government subsidy (Sub), enterprise profit (Profit), and operating revenue (Revenue) are used as mediating variables to explore further the mechanism of centralized band purchasing policy affecting enterprise innovation. Drawing on the study of Jiang et al. (46), the amount of government subsidies received by the enterprises in the sample in the current year is used as a measure of government subsidies (Sub); drawing on the study of Zhu (47), the gross profit margin (the ratio of the profit from the main business divided by the revenue from the central business) is used to measure the profit of the enterprise; drawing on the study of Liu (48), the operating income of the enterprise in the current period (in billions of Yuan) is used as a measure of the operating income (Revenue). The operating income (Revenue) is used as an intermediary variable. Revenue is used as the indicator of business income.

Referring to the research of Jiang (49), in order to avoid the problem of excessive use of the stepwise method of mediated effects to test the transmission mechanism, the third step of the operation of estimating the size of the indirect effect and testing the statistical significance is canceled, and the following regression model is established:


(2) $$Mech_{i,t}=\alpha_{0}+\alpha_{1}Post_{i,t}\times Treat_{i,t}+\phi X_{i,t}+\mu_{i}+\gamma_{i}+\varepsilon_{i,t}$$

In Eq. (2), the mechanism variable Mech uses three proxies of “government subsidy (Sub), enterprise profit (Profit) and operating income (Revenue)” in turn. The rest of the symbols have the same meaning as in Eq. (1).

The regression results of the mechanism of influence on the innovation of pharmaceutical enterprises of the centralized band purchasing policy of drugs are shown in Table 10. Column (1) shows the regression results of Sub as a mediating variable. The coefficient of variable Post ×Treat is 0.8491 and is significant at the 5% level, indicating that the centralized banded purchasing policy for medicines can promote enterprises to obtain higher government subsidies. Government subsidies provide an incremental source of funds to alleviate the resource constraints faced by enterprises’ innovation, reduce the marginal cost of their innovation activities, and diversify the risks. Guo et al. (50) argue that the actual benefits of enterprises engaged in R&D activities are smaller than the total social benefits, and government subsidies can compensate for the costs of enterprises, which has a better positive incentive effect on enterprise innovation. This effect shows heterogeneity in industry and regional space. Huang et al. (51) argued that government subsidies as a public policy can promote enterprises to carry out innovative activities in terms of technology and products, etc. After obtaining government subsidies, enterprises will enhance their technological innovation capacity through the means of research and development of new products, the introduction of new technologies, equipment, and processes, etc., which will, in turn, lead to the enhancement of economies of scale, thus improving their innovation performance. Therefore, the centralized band purchasing policy for medicines can promote enterprise innovation by facilitating government subsidies. Thus, H2 is verified.

**Table 10:** Mediation effect test results.

	Sub	Profit	Revenues
Post×Treat	0.8491^**^	0.0317^**^	0.5350^*^
	(0.3739)	(0.0157)	(0.3138)
_Cons	−40.9040^***^	0.7472^*^	−49.9423^***^
	(9.6047)	(0.4323)	(13.9845)
Control variable	Yes	Yes	Yes
Individual effect	Yes	Yes	Yes
Time effect	Yes	Yes	Yes
N	924	924	924
Adj. R2	0.1755	0.1083	0.3469

^*^, ^**^, ^***^ indicate significant at the 10 per cent, 5 per cent and 1 per cent levels, respectively, with standard errors in parentheses.

Column (2) in Table 10 shows the regression results of Profit as a mediating variable, and the coefficient of the variable Post × Treat is 0.0317 and is significant at the 5% level, indicating that the centralized band purchasing policy of medicines has a positive impact on corporate profits. Introducing this policy makes pharmaceutical enterprises drop to optimize production and operation activities, reduce the cost of enterprise operation, effectively use funds, and improve the Profit of enterprise operation. Zhang et al. (52) argued that the improvement in the profits of pharmaceutical companies has stimulated their motivation to supply innovative drugs, thus reducing the risk of shortage of innovative drugs. Gu and Qi (53) argue that the financial environment impacts corporate innovation. In the high economic upturn stage, enterprises’ profit levels are higher, and the financing environment is more favorable, which provides good financial support for enterprise innovation activities. Therefore, the centralized band purchasing policy for medicines can promote corporate innovation by increasing profits. Thus, H3 is verified.

Column (3) in Table 10 shows the regression results of Revenue as a mediating variable, and the coefficient of the variable Post × Treat is 0.5350 and is significant at the 10% level, which indicates that the centralized band purchasing policy of medicines has a positive impact on the business revenue of enterprises. With the increase in business revenue after introducing this policy, the enterprise’s profitability has been significantly improved. At the same time, the timely return of the government has a positive impact on the enterprise’s financial status, prompting an increase in cash flow and providing stable financial support for the development of the enterprise. Adequate free cash flow is essential for enterprises to conduct research and development activities. Wang et al. (54) argue that free cash flow plays a crucial role in connecting corporate R&D activities to the capital market. Wang et al. (55) found that an increase in the net cash flow generated by an enterprise’s operating activities will help support R&D and innovation activities and further increase the intensity of the enterprise’s investment in technological innovation. Therefore, the centralized band drug purchasing policy can promote corporate innovation by increasing operating income. Thus, H4 is verified.

## 7 Conclusions and implications

The centralized band purchasing policy is an integral part of China’s drug review and approval system reform and the promotion of the pharmaceutical and health system reform. It plays a vital role in lowering the price of medicines, improving the quality of medicines, guiding healthy competition in the pharmaceutical market, and stimulating the innovation and development of pharmaceutical enterprises. In this paper, the implementation of the centralized band purchasing policy of drugs is regarded as a quasi-natural experiment, and the impact of this policy on the innovation of pharmaceutical enterprises and its mechanism of action is empirically analyzed using the double difference method. The conclusions are as follows: (1) The centralized band purchasing policy significantly impacts pharmaceutical enterprise innovation. (2) The impact of the centralized band purchasing policy on the innovation of pharmaceutical enterprises is heterogeneous regarding region, enterprise size, and nature of equity. Regarding different regions, the policy can only promote the innovation of pharmaceutical enterprises in the eastern region, and the effect on the central and western regions is insignificant. Regarding firm size, the policy promotes innovation inputs only for small and medium-sized enterprises and promotes innovation outputs of large firms more than for small and medium-sized enterprises. Regarding the nature of different equity, the policy only promotes the innovation input of non-state-owned enterprises, and its role in promoting the innovation output of state-owned enterprises is more vital than that of private enterprises. (3) The centralized band purchasing policy promotes innovation in pharmaceutical enterprises by increasing government subsidies, corporate profits, and operating income.

Based on the above findings, this study makes the following recommendations:

1. Strengthening the orientation of centralized band purchasing of medicines to promote enterprise innovation and guiding and encouraging pharmaceutical enterprises to invest in technological innovation and research and development based on the centralized band purchasing of medicines policy. It is also necessary to recognize the heterogeneity of the centralized band purchasing policy in promoting technological innovation among enterprises in different regions, of different enterprise sizes, and different ownership. In implementing the policy, it is necessary to consider efficiency and balance and accelerate the realization of the goals of promoting technological innovation and enterprise development to ensure that the centralized band purchasing policy of medicines has the best effect. Specifically, the government should take complete account of the actual differences in the situation of pharmaceutical manufacturers in the eastern, central and western regions. It should increase the support for the innovation system in the central and western regions and improve innovation in the western and central areas by optimizing the facilitation of investment in innovation, the introduction of talents, and the strengthening of the implementation of the foundation with the support of the centralized band purchasing policy of medicines; and at the same time, it should set up cross-region innovation alliances or cooperation platforms to promote the resource sharing and collaborative innovation in the face of the previously existing differences between large pharmaceutical producers and small and medium-sized pharmaceutical producers. The government should guide SMEs with innovation and competitiveness to develop in the industry and adjust the flexibility of the policy to enrich the diversified market demand by reserving a certain percentage of the market share for eligible enterprises. The government can stimulate large enterprises to increase their R&D investment in original research and development (ORD) drugs through innovation subsidies and technical support. It can also promote the transition from producing generic drugs to ORD drugs to promote competitiveness and innovation in the drug market. Secondly, the government should consider the different behaviors of enterprises with varying property rights in the face of centralized band purchasing drugs. The government should promote the transformation of state-owned enterprises, stimulate the innovation vitality of state-owned enterprises, incorporate technological innovation into employee performance appraisal, and promote the innovative behavior of state-owned enterprises. The innovation of enterprises needs a lot of money, and the government should formulate a comprehensive science and technology subsidy policy to increase the financial support for the technological innovation of enterprises so as to reduce the pressure faced by enterprises in the process of innovation.
2. Against the backdrop of the normalization and institutionalization of the centralized band purchasing policy for medicines, if enterprises want to stand out in the highly competitive market, they must realize the importance of innovation, take innovation as the core content of their corporate strategy, devote themselves to the research and development of medicines with innovative targets or mechanisms, and resolutely resist unethical competition in order to build a pharmaceutical enterprise with superior R&D strength. Large enterprises should make full use of their rich technical experience, continuously develop new technologies based on existing technologies, improve the level of research and development, and enhance the core competitiveness of enterprises in the industry; small and medium-sized enterprises should increase investment in research and development, the introduction of advanced equipment and technology; increase the introduction of talents, cultivate high-quality R & D team, and at the same time, cooperate closely with universities and research institutes to promote the improvement of R & D level and enhance the core competitiveness of enterprises; and always pay attention to industry trends, grasp the cutting edge of innovation in the industry. They should also cooperate with universities and research institutes to promote the improvement of the R&D level, enhance the core competitiveness of the enterprises, pay attention to the industry trends, and grasp the innovation frontier of the industry. State-owned enterprises should combine their conditions, give full play to their advantages, dare to “go out” and actively carry out innovative activities, have an in-depth understanding of the market frontier, constantly learn and absorb advanced technical knowledge, enhance the sense of innovation, and strengthen the R & D capabilities. Non-state-owned enterprises should conform to the development of national strategies, actively change their development strategies, constantly explore and innovate, rely on innovation and quality to win the market and increase the added value of their products, avoid homogenized competition and develop diversified products to meet the diversified needs of consumers.
3. Use more targeted policy tools to help the centralized band purchasing policy for medicines to be implemented in a high-quality manner. The results of the empirical study show that the centralized banded procurement of medicines policy promotes the innovation input and output of pharmaceutical enterprises by increasing government subsidies, corporate profits, and operating revenues. Therefore, the formulation of relevant supportive policies to promote enterprise government subsidies, enterprise profits, and operating income can further enhance the role of the centralized band purchasing policy on pharmaceutical enterprise innovation. For example, by developing reasonable R&D and innovation of financial policy leadership, the government should strengthen the service guarantee, focusing on promoting enterprise innovation. At the same time, it also needs to guarantee adequate funds to help enterprise innovation.

This study still has several areas for improvement, which may also be worth further exploring. First, only Chinese A-share listed companies in Shanghai and Shenzhen were selected as the research object, and the impact of the centralized bulk drug purchasing policy on the innovation behaviour of unlisted companies was not considered. In the future, the innovation impact of the centralized band purchasing policy of drugs on unlisted pharmaceutical manufacturing enterprises can be further explored. Second, this paper needs to control for the impact of other policies (e.g., generic consistency evaluation) on firms’ innovation during the sample period, resulting in a lack of rigour in the paper’s analytical results. Subsequent studies can add other policy control variables to increase the scientificity and rigor of the study. Third, this paper only analyzes the mechanism from the perspectives of government subsidies, corporate profits and operating revenues. Still, in practice, there may be other factors (e.g., market competition, etc.) as a mechanism to affect corporate innovation, so it is necessary to explore the mechanism analysis more deeply in the future.

## Data availability statement

The datasets presented in this study can be found in online repositories. The names of the repository/repositories and accession number(s) can be found in the article/supplementary material.

## Author contributions

XC: Conceptualization, Data curation, Formal analysis, Investigation, Methodology, Project administration, Validation, Writing – original draft. SC: Conceptualization, Writing – original draft. ZW: Validation, Writing – original draft. SW: Supervision, Writing – review & editing. YC: Supervision, Writing – review & editing.

## Footnotes

^1^ http://cndata1.csmar.com/

^2^ http://www.cninfo.com/
